# Impact of a Social Media Campaign on Reach, Uptake, and Engagement with a Free Web- and App-Based Physical Activity Intervention: The 10,000 Steps Australia Program

**DOI:** 10.3390/ijerph16245076

**Published:** 2019-12-12

**Authors:** Anna T. Rayward, Corneel Vandelanotte, Kelly Corry, Anetta Van Itallie, Mitch J. Duncan

**Affiliations:** 1 School of Medicine & Public Health, Faculty of Health and Medicine, The University of Newcastle, University Drive, Callaghan, NSW 2308, Australia; Anna.Rayward@uon.edu.au (A.T.R.); mitch.duncan@newcastle.edu.au (M.J.D.); 2 Priority Research Centre for Physical Activity and Nutrition, The University of Newcastle, University Drive, Callaghan, NSW 2308, Australia; 3 Physical Activity Research Group, Appleton Institute, Central Queensland University, Bruce Highway, Rockhampton, QLD 4700, Australia; k.corry@cqu.edu.au (K.C.); a.vanitallie@cqu.edu.au (A.V.I.)

**Keywords:** physical activity intervention, social media, e-Health, recruitment

## Abstract

Social media campaigns provide broad-reach and convenience for promoting freely-available health programs. However, their effectiveness and subsequent engagement of new users is unknown. This study aimed to assess the reach and new member registration rates resulting from a dedicated 10,000 Steps social media campaign (SMC) and to compare program engagement and time to non-usage attrition of new users from the SMC with other users. SMC reach (using Facebook, Instagram, and display advertisements engagement metrics), new-user numbers, engagement (usage of the website and its features), and time to non-usage attrition were assessed using generalized linear regression, binary logistic regression, and Cox proportion hazards regression models. During the SMC, Instagram and display advertisement impressions, Facebook reach and new daily registrations were significantly higher compared with six weeks and one year prior. There were no between-group differences in the average usage of most website/program features. Risk of non-usage attrition was higher among new users from the SMC than new users from one year prior. The SMC was effective in promoting awareness of the 10,000 Steps program. Further research to identify long-term engagement strategies and the most effective combination of social media platforms for promotion of, and recruitment to, health programs is warranted.

## 1. Introduction

Insufficient physical activity is the fourth leading risk factor for mortality worldwide [1], with 23.8% of the global population being physically inactive [2]. Therefore, effective, broad-reaching and cost-efficient programs to promote physical activity are required. With internet access now widely available (86% of Australian households are connected, and 56% of the global population are active internet users) [3,4], internet-delivered programs provide broad reach and produce positive changes in physical activity [5]. The 10,000 Steps program is a free, publicly available, internet-delivered program to promote physical activity, which began in 2001 [6] and, as of December 2019, had over 425,000 users.

Common methods of promoting the program have included traditional advertising (e.g., newspaper, radio), the use of health professionals, optimizing its location in online searches (e.g., use of keywords to increase the likelihood it is found in online searches), word-of-mouth, and promotion within workplaces and communities. However, since the advent of the 10,000 Steps program, a range of new promotional options have become available through the development of online social media.

Social media is a group of internet-based applications that allow communication and the exchange of user-generated content [7]. Popular social media websites include Facebook and Instagram, with 15 and 9 million users in Australia, respectively [8]. One significant benefit of applying social media tools to promote health programs is that promotional content can be tailored and targeted towards specific populations, making it much easier to fill gaps in the reach of programs [9]. Several systematic reviews examining the use and success of Facebook to recruit participants to health-related studies concluded that this method was superior compared to traditional recruitment methods in terms of lower costs, faster recruitment times, and recruitment of people with demographic characteristics more representative of the general population, other than an over-representation of white, young females [10,11]. Yet, another review indicated that only half of the studies using social media for recruitment found it to be the most effective strategy [12]. It is, however, important to emphasize that many of these studies were recruiting participants into controlled trials or cohort studies which differ in their scope, expectations, and requirements of participants in comparison to freely available health promotion websites such as 10,000 Steps. Therefore, whilst social media platforms have the potential to be powerful tools for advertising health promotion interventions, such as the 10,000 Steps Program, their effectiveness is not yet firmly established.

In January 2018, a six weeks social media campaign (SMC) to promote the 10,000 Steps program was launched using Facebook, Instagram, and display advertisements, in an attempt to increase the number of individuals registering with the 10,000 Steps program. Evaluation of the reach of this social media campaign and examining how this reach translates into improved uptake of the program is important for understanding the merit of utilizing this form of promotion in the future. Whilst several studies have indicated that social media can successfully reach participants in high volumes [12,13], it is not known if these new program members engage with the program in a meaningful way or whether promotion simply compels people to register but then not engage with the program. For example, a previous study found social media to be a more cost efficient recruitment strategy compared to traditional methods but participants recruited via social media for were less likely to complete the study, suggesting a lower level of conscientiousness [14]. Therefore, the aims of this study are to determine the reach of this 10,000 Steps social media campaign, assess new member registration rates as a consequence of the social media campaign and compare program engagement and time to non-usage attrition of the new members resulting from the social media campaign with 10,000 Steps users attracted using different methods.

## 2. Materials and Methods

### 2.1. Participants and Procedures

The 10,000 Steps program is a free, publicly available program utilizing multiple strategies to encourage adult participants to increase their physical activity. The program was developed using a socioecological framework and began as a whole-community program in Rockhampton, Australia [6]. Details of the development of the program are available elsewhere [15]. When registering with the 10,000 Steps program, participants provided informed consent for the usage of their data for research purposes.

A key component of the program is the accumulation and self-monitoring of physical activity [16]. Users are able to monitor their daily physical activity levels by recording their pedometer/activity tracker-counted steps and/or minutes of moderate and vigorous physical activity using an online step log. The online step log is available to users on both the 10,000 Steps website and a smartphone app. Activity and/or steps logged using the app are automatically synced with the user’s account and any activity and/or steps a user has logged using the website [17]. Additional features available to users include monthly Challenges for individuals (users may choose from a selection of virtual “walking journeys” with predefined monthly step goals and receive graphical and text-based feedback in relation to progress), team Tournaments (team-based virtual walking challenges based on a set amount of time or a predefined journey and created by 10,000 Steps Coordinators—workplaces, community organizations, groups of friends), and virtual friends (which allow users to connect and view one another’s progress to keep each other motivated) [17].

Participants were new users of the 10,000 Steps program, from Queensland, who registered during a social media campaign (21 January 2018 to 3 March 2018), during the six weeks prior to this social media campaign (10 December 2017 to 20 January 2018) and who registered during the same period one year prior to this social media campaign (21 January 2017 to 3 March 2017). The two comparison groups covered a proximal time-point and an equivalent time-point one year prior to this social media campaign with which to compare sign–up rates and to account for natural seasonal variation in registrations. Only the comparison group from one year prior to this social media campaign was used to compare engagement metrics since the six weeks prior to the social media campaign included the Christmas/New Year period and the potential for disruption to users’ normal routines, which may limit comparisons of engagement between the two periods.

### 2.2. Social Media Campaign

**Figure 1:**
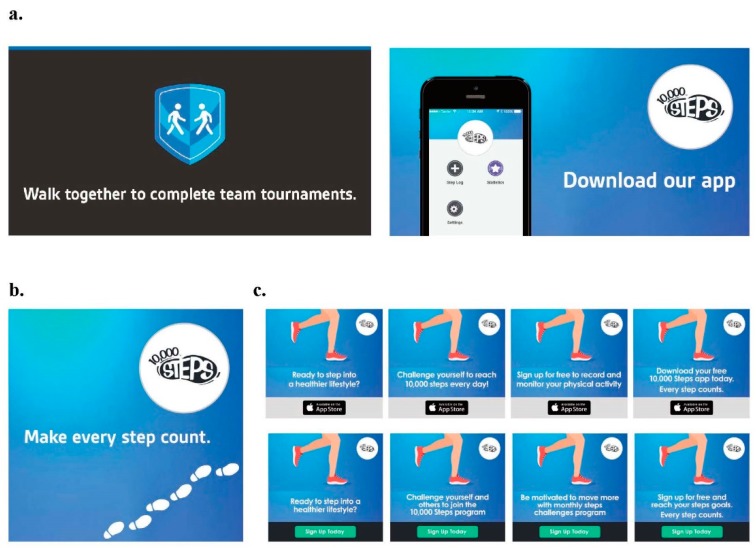
(**a**) Facebook advertisement examples; (**b**) Instagram advertisement example; (**c**) mobile display advertisement examples.

A social media campaign (SMC) was initiated by the 10,000 Steps program, facilitated by CQ University’s online marketing team and implemented by an external company. This social media campaign was via paid ads in addition to organic content on the 10,000 Steps Facebook page. This social media campaign targeted individuals in Queensland Australia and ran for six weeks from 21 January 2018 until 3 March 2018. Three digital media platforms were utilized. Paid Facebook advertisements targeted two different audiences: Office Workers and a Health and Fitness Audience (those who had expressed interest in health and fitness Facebook content). These Facebook advertisements were delivered on the 10,000 Steps page aiming to enhance awareness of the program and drive visitation to the website and subsequent registration with the program. Due to 10,000 Steps not having their own account at the time, CQ University’s Instagram account was used to deliver ads about this program with the aim to provide broad awareness of the campaign. Mobile display advertisements (e.g., an advertisement banner displayed at the top or side of a website page on internet-enabled devices including mobile devices) were used to further raise awareness of the program, website, and app (Figure 1).

### 2.3. Data Collection and Extraction

Overall daily and total Facebook reach, engagement and likes, Instagram impressions, and display advertisements were assessed using data from the platforms. These data were at the aggregate level for all social media users irrespective of their geographical location, and data for the target region of Queensland were not available.

Measures of user characteristics, engagement, and platform usage were extracted from the 10,000 Steps website database and Google Analytics, as detailed below. For the social media campaign group, usage and engagement data for new users were tracked from the time of registration until the 31 December 2018. For the year prior comparison user group, usage and engagement data for new users were tracked from time of registration until 31 December 2017. The related data are available in Supplementary Tables S1–S3.

## 3. Measures

### 3.1. User Characteristics and 10,000 Steps Program Registrations

Date of birth, gender, and registration date were recorded and stored in the website database. Date of birth was used to determine age at time of registration. Gender was categorized as male, female, or other.

New Queensland-based users of the 10,000 Steps program were assessed using three time-points based on their registration date: during the 6 weeks social media campaign period (21 January 2018 to 3 March 2018), during the six weeks prior to the social media campaign (10 December 2017 to 20 January 2018), and during a 6-week period one year prior to the social media campaign (21 January 2017 to 3 March 2017).

### 3.2. Facebook, Instagram, and Display Advertisement Costs and Parameters

Social media campaign costs included promotion asset and copy development costs and advertisement delivery costs. Facebook, Instagram, and display advertising were assessed as total cost, cost per click, and cost per thousand impressions.

Facebook reach was assessed as the average daily and total number of unique users who were exposed to any content from, or about, the 10,000 Steps Facebook page on their screen, including posts, check-ins, ads, and social information from people who interacted with the page during the social media campaign. Facebook page engagement was assessed as the average daily and total number of unique users who engaged with the 10,000 Steps Facebook page, including any clicks, comments, or shares. Facebook page likes were assessed as the average daily and total number of new unique users who liked the 10,000 Steps Facebook page. Instagram reach was assessed using the total number of impressions (number of single views of an ad by one individual) across the social media campaign period. Display advertisements reach was assessed by impressions for each of iOS, Android, and website media.

### 3.3. User Engagement with Website and Program

Users’ engagement with the 10,000 Steps website was assessed using average number of sessions, average number of pages viewed per session, average number of step entries, and average daily step count. Participation in individual Challenges and team Tournaments, connecting with Friends, and ever logging steps were all dichotomized to “yes” or “no” (yes = participated in ≥1 Challenge or Tournament; yes = received or sent ≥1 friend request; or yes = logged steps ≥1). Time to non-usage attrition (classified as no step entries for 14 consecutive days) was calculated using data exported from the website, similar to previous studies [18].

## 4. Statistical Analysis

Results were expressed as differences in the least square group means with 95% CIs, and ORs and HRs with 95% CIs. Alpha was set at 0.05 for all analyses, which were conducted using Stata V15.1. in April 2019 [19]. User characteristics, Facebook, Instagram, and display advertisement costs and metrics were described using descriptive statistics.

To examine differences in Queensland registrations to the 10,000 Steps program between the social media campaign time period and the six weeks prior to the social media campaign and the same time one year prior to the social media campaign, a generalized linear regression with negative binomial family and identity link function was used.

Differences in engagement for website sessions, pages per session, daily step count, and total step entries between Queensland users who registered during the social media campaign (SMC group) and Queensland users who registered during the equivalent time period one year prior (Year Prior group), were examined using generalized linear regression models for those with data for each outcome. Negative binomial family and identity link functions were used for all analyses except step count, which used a Poisson family and identity link model. Residual diagnostics informed the specification of family and link functions. All models were adjusted for participation in Challenges and Tournaments.

Binary logistic regression was used to examine associations between the SMC and Year Prior groups in engagement via participating in Challenges and Tournaments, receiving and sending friend requests, and ever logging steps. These models were adjusted for Challenges and/or Tournaments depending on the outcome examined (except ever logging steps) and are specified in the relevant table.

Participating in the online Challenges and Tournaments has been shown to influence usage and engagement with the 10,000 Steps program [17,20]. Therefore, between-group differences in time to non-usage attrition were examined using two Cox proportion hazards regression. First, in an unadjusted model, and second, also adjusting for participation in Challenges and Tournaments. The time to non-usage attrition was plotted using Kaplan–Meier survival estimates and adjusted survival curves. Schoenfeld residuals were examined, and no obvious violations were observed.

## 5. Results

A total of 1242 new Queensland users registered with the 10,000 Steps program during the social media campaign period, 210 new Queensland users registered during the six weeks prior to the social media campaign, and 415 new Queensland users registered during the same time the year prior (Table 1 and Table 2). The mean age was 44 years, and 78% were female (Table 1). Overall, among the SMC and Year Prior groups, from the time of registration until the end of their registration year (31 December 2018 and 2017, respectively), 84% engaged with the website at least once based on making at least one step entry, and an average of 9626 daily steps were logged. On average, these users engaged with the website 17 times (sessions) and viewed nine pages per session (Table 1).

### 5.1. Cost and Reach of Social Media Campaign

The total cost of the social media campaign was AUD $29,062 ($4950 for advertisement development; $24,111 for advertisement delivery). The costs for each of Facebook, Instagram, and display advertisement delivery were: $11,668, $5000, and $7500, respectively (Table 3). Costs per click and costs per thousand impressions for each medium are shown in Table 3.

The total number of people exposed to any Facebook content regarding the 10,000 Steps program (reach) during the social media campaign was 391,131. Total Facebook reach in the six weeks prior and same period one year prior was 5266 and 11,591, respectively. Instagram and display advertisement impressions totalled 819,192 and 577,023 during the social media campaign (Table 2).

### 5.2. Differences in Daily Registrations Between Different Time Periods Relating to Social Media Campaign

A statistically significantly higher number of daily new Queensland users of the 10,000 Steps program registered during the social media campaign period compared with both the six weeks prior to the social media campaign (29.6 vs. 5.0 per day, *p* < 0.001) and the same time one year prior to the social media campaign (29.6 vs. 9.9 per day, *p* < 0.001) (Table 4).

### 5.3. Between-Group Differences in Engagement with the 10,000 Steps Program

The Year Prior group users were 57% less likely to participate in individual Challenges, nearly four times more likely to participate in team Tournaments, and nearly three times more likely to have logged steps at least once than the SMC group users from the time of registration until the end of their registration year (31 December 2018 and 2017, respectively) (Table 5). There were no between-group differences in the average number of sessions, pages viewed per session, daily step count, total step entries or likelihood of receiving or sending friend requests (Table 6).

### 5.4. Program Engagement

**Figure 2:**
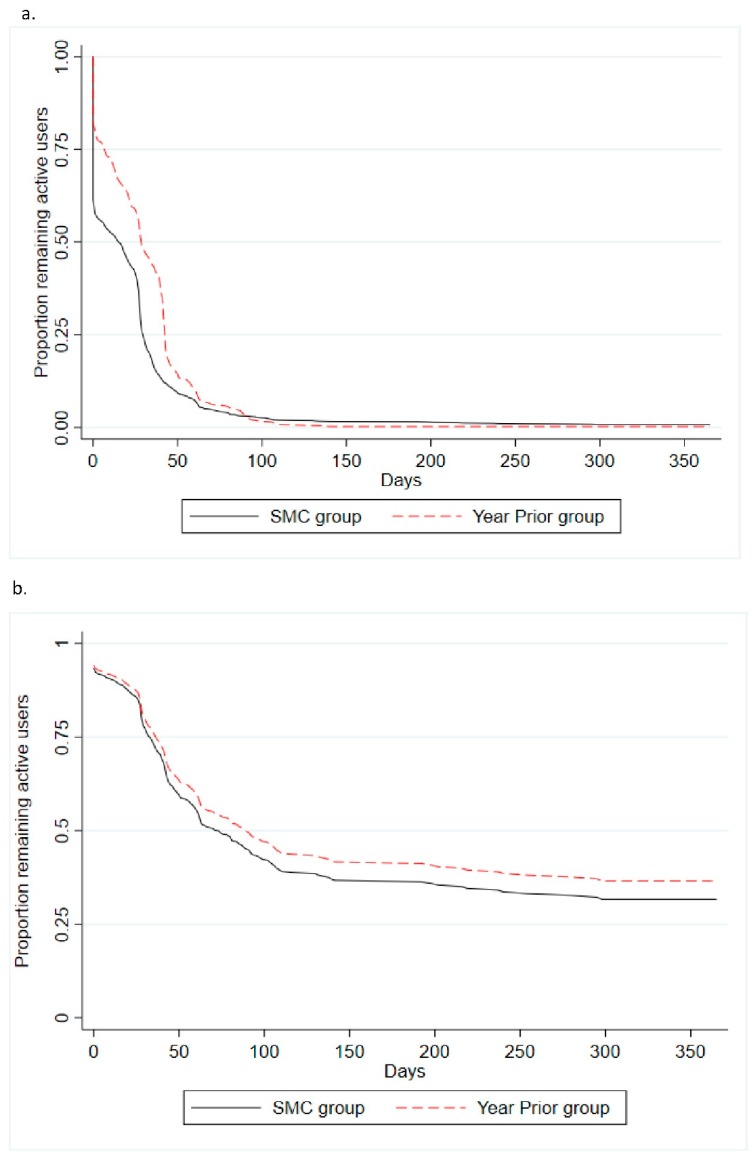
(**a**) Kaplan–Meier estimates of the survival distribution for time to non-usage attrition by group; (**b**) Cox proportional hazards regression curve of the survival distribution for time to non-usage attrition by group based on the model adjusted for Challenges and Tournaments (Table 7; HR = 1.14, 95% CI: 1.01, 1.28).

From the time of registration until 31 December in the same year (2018 for SMC group and 2017 for Year Prior group), steps were logged at least once by 1104 new users (SMC group = 764 (61.5%); Year Prior group = 340 (81.9%) (Table 1). A total of 1647 (99.4%) of all users succumbed to non-usage attrition (SMC group = 1233 (99.3%); Year Prior group = 414 (99.8%)) with the average time to attrition being 25 (±40) days (SMC group = 23 (±42); Year Prior group = 31 (±30)). The SMC group had a 39% higher risk of succumbing to non-usage attrition than the Year Prior group (HR = 1.39; 95% CI: 1.24, 1.56) (Table 7), and the difference between groups remained following adjustment for participation in Challenges or Tournaments although this difference was slightly attenuated (HR = 1.14; 95% CI: 1.01, 1.28) (Table 7). Users who did not participate in Challenges or Tournaments had 3.7 and 3.02 times higher risks, respectively, of succumbing to non-usage attrition than users who did, irrespective of their group (Table 7). Kaplan–Meier survival estimates and adjusted Cox proportional hazards regression curves for step entry are shown in Figure 2. Estimated median lifetime usage (time after which 50% ceased logging steps) for all users was 21 days (Table 8).

## 6. Discussion

This social media campaign conducted by the 10,000 Steps program reached a relatively large number of individuals. There was a concomitant increase in the numbers of people registering with the program during the social media campaign, which was significantly greater than during both the six weeks prior to the social media campaign and the same time period as the social media campaign one year earlier. However, fewer new users resulting from the social media campaign started using the website and app, and they were more likely to stop logging steps earlier than users who joined the program a year earlier. Though importantly, when the new users who registered during the social media campaign engaged with the website, they logged steps in much the same manner as other users of the program. These findings indicate that using social media platforms provides a broad-reaching, convenient, and effective method to enhance awareness and uptake of freely available physical activity programs, although users reached by a social media campaign may disengage with programs sooner than other users.

### 6.1. Social Media Campaign Reach and Cost

The three forms of social media utilized in the campaign produced nearly 1.9 million impressions overall. Facebook advertising resulted in a 33-fold increase in the number of people who were exposed to information about the 10,000 Steps program and a 16-fold increase in engagement with the 10,000 Steps Facebook page, compared with the same time the year before. Mobile display advertisements were viewed more than half a million times, whilst Instagram advertisements reached the largest audience by far, being viewed over 800,000 times. Since alternative ways to “find” the 10,000 Steps program existed concurrently during the social media campaign, there is no way of knowing how many new registrations were directly attributable to exposure to this social media campaign. However, the large number of impressions is likely to have heightened awareness of the program, with interested people able to search for the website of their own volition. Furthermore, despite the social media campaign targeting Queenslanders (which nearly tripled Queensland registrations), there was a substantial spike of nearly an additional 50% of new registrations beyond Queensland residents (Table 2), indicating that social media provided additional reach outside the initial target area. This raises the question of the need to limit social media campaigns for widely-available health promotion programs to local areas given the global nature of the internet. Regardless, the ability of social media to disseminate messages about physical activity and the availability of free programs such as 10,000 Steps, on a vast scale, with a significant associated increase in new registrations, as demonstrated by this social media campaign, indicates that this form of awareness-raising is a valuable tool for health promotion projects.

It is difficult to compare numbers of impressions between different social media advertising campaigns since they can vary by overall recruitment timeframe, target audience, advertisement delivery scheduling, and expenditure [10,21]. Additionally, most previous research has examined recruitment for clinical trials, whereas the campaign in this study was designed to promote awareness of a long-running free, publicly available program, without specific recruitment targets. Previously reported social media recruitment costs for RCTs with restrictive eligibility criteria have varied widely; however, one review reported an average costs/participant ranging from USD $1.36 to USD $76.15 (mean = USD $19.97). Acknowledging that not all of the registrations during the social media campaign are directly attributable to the campaign, the cost per registrant (for this study was USD $13.67 (AUD$23.40). This indicates a relatively favourable comparison to other social media campaigns considering the campaign was broad and the lack of “click through” (when someone clicks on an advertisement and a new website window opens) capacity of Instagram, which was the medium responsible for the greatest exposure. It also compares favourably to traditional media costs, which averaged USD $38/participant [12].

The click through rate was not particularly high. However, allowing potential users to click through to the 10,000 Steps website, effectively overcomes the time lapse gap between becoming aware of the program and the opportunity to sign up, during which time interest may be lost or other priorities take over. While not available at the time of this social media campaign, Instagram has since added the capacity to add a link to click through to a website. Given that Instagram achieved by far the largest number of impressions of the media used (more than double the impressions achieved by Facebook), this new functionality may be even more beneficial in creating awareness and directing traffic to the website for new registrations. Much of the research into the use of social media for promotion of health programs or trials has focused primarily on Facebook [10,14,21]. Although the social media landscape keeps evolving, further research, examining the most effective, or combination of, social media platforms for promotion of, and/or recruitment to, health programs, may enable researchers and health promoters to better plan cost-effective, efficient, high-number engagement and recruitment strategies.

The typical age of those recruited to health-related studies via social media is 16–24 years, although there tends to be a preponderance of studies using Facebook to target younger rather than mid-to-older adults [10]. This study found new users to be middle-aged on average. This may be a reflection of substantial proportion of mid-to-older adults who engage with social media (69% of those aged 45 to 54 years and 60% aged 55 to 64 years) [22] and that adults aged 45 to 54 years have greater awareness of the 10,000 Steps program than 18 to 43-year-old adults [23] or the typical demographic of the program [17]. Social media statistics indicate that Facebook is the most popular social media platform for Australian users [24]. Australians aged 29 to 73 years most commonly use Facebook (~87%), followed by YouTube (65–82%) and Instagram (15–52%) [24]. Meanwhile, those aged ≤28 years most commonly use YouTube (89%) followed by Facebook (82%) and Instagram (68%) [24]. Therefore, this study provides new evidence that social media may be useful for promotional purposes across a broader range of age groups than previously thought [10].

### 6.2. Queensland Sign-Up Rates Resulting from Social Media Campaign

Programs that are run in real-life settings and over the long term need to employ new strategies to attract new users. The social media campaign resulted in a six-fold increase in the number of new Queensland registrations compared to the six weeks prior, and a three-fold increase compared to the same time the year prior. Therefore, it is clear that capitalizing on the 89% of Australian adults who have internet access, 15.2 million (79%) who use mobile devices [22], and the millions of people who use Facebook and Instagram [8] is advantageous for attracting people to publicly-available health programs.

It is difficult to compare the success of this social media campaign with other studies since most others aim to recruit participants for randomized controlled trials with specific target numbers and eligibility criteria, whereas this campaign aimed to promote engagement with of an existing health program [10,21]. However, the 1242 new registrants to the 10,000 Steps program is equivalent to some large sample sizes recruited for other studies using social media [10,13] and larger than the number of people (n = 205) who reached a web-based physical activity randomized controlled trial (RCT) program via Facebook advertising prior to being screened for eligibility (and therefore more representative of the effectiveness of social media advertising to attract people to real-world web-based physical activity programs) [11].

### 6.3. Engagement with the 10,000 Steps Program

The concern with acquiring new users via a social media campaign is that after being stimulated to register, subsequent meaningful engagement with the program may not occur in the same way they may have if they were introduced to the program otherwise (e.g., as part of a workplace initiative). Previous research has shown that despite social media campaigns having the capacity to increase the volume of recruits [12], the rate of completion of health studies (drop-out attrition) was less than for those recruited using traditional methods and this may also reflect a lower level of engagement with the studies [14]. In this study, people recruited during the social media campaign who used the website on average had similar rates of engagement with the website to those who registered the year prior. However, a larger proportion of the SMC group used only logged steps once and were more likely to succumb to non-usage attrition and do so more quickly (Table 8; Figure 2). These results suggest that although social media campaigns can be useful to get people initially registering and using the program, they engage with the program in different ways. The high attrition rate might also be related to the lack of a completion date in a real-world health program, which may otherwise provide motivation to continue engaging with the program for longer. This may require using tailored strategies to help these users be exposed to sufficient content from the program to promote behaviour change. For example, users may be prompted to participate in program components that have previously been shown to increase engagement with the 10,000 Steps program, such as Challenges or similar leader-board functionality on the website [17,20]. Additionally, these program components utilize effective behaviour change techniques, such as goal-setting, self-monitoring, and feedback [25,26].

Few differences in program usage were observed. The Year Prior group were less likely to participate in individual Challenges but more likely to engage in team Tournaments. This may be due to many of that group being part of workplace initiatives, which are a major component of the general promotional strategies employed by 10,000 Steps. Users who are engaged with Tournaments may be less likely to go looking for other motivational features such as the Challenges. Conversely, new users resulting from the social media campaign were likely to have started the program as an individual rather than as part of a workplace initiative. Therefore, they have less opportunity to participate in a team Tournament since Tournaments require a dedicated coordinator for the organization. The Challenges and Tournaments are both strategies to improve motivation to increase step counts. Since both groups reported similar step counts, it would seem that these differences in the likelihood of participating in Challenges and Tournaments compensate for one another and do not translate to lower or higher step counts in either group.

### 6.4. Time to Non-Usage Attrition

The SMC group was more likely to succumb to non-usage attrition earlier than the Year Prior group (23 days versus 31 days). Participation in Challenges and Tournaments greatly reduced the risk of non-usage attrition in both groups. A smaller proportion of users from the SMC group participated in Tournaments and Challenges and consequently stopped logging steps sooner than the Year Prior group.

This finding aligns with a previous study examining a group of 10,000 Steps program users. That study found that workplace challenges (now called team Tournaments) were associated with a reduced risk of attrition [17]. Despite this, the SMC group time to non-usage attrition was much longer than a previous real-world study involving the 10,000 Steps program [27] and less than a third shorter than an app-based physical activity and sleep RCT, which had the advantage of participants being actively recruited to a program with a more formal structure [28]. Therefore, whilst the social media campaign significantly improved registration numbers to the 10,000 Steps program, strategies which promote longer engagement with the program should be explored. Low participant engagement has long been a problem for mhealth interventions [29], and some strategies that are effective at improving engagement, such as face-to-face intervention components [30], are not necessarily feasible in eHealth/mHealth or large scale programs [31]. Engagement in eHealth/mHealth interventions is influenced by several factors including the content of the intervention itself where particular behaviour change strategies appear to be useful to increase engagement (i.e., action planning, social support features, self-monitoring tools, reminders). Previous eHealth/mHealth research has suggested that strategies, such as increasing program interactivity and generating online social networking and support, may enhance engagement [31]. Therefore, stronger promotion of participation in Challenges and Tournaments may provide greater interactivity, encourage the development of social networks, and generate more sustained program engagement.

### 6.5. Strengths and Limitations

A strength of the study is the examination of the utility of social media for promotion of a publicly available, real-world program. Most studies have evaluated its utility for recruitment in RCTs and, therefore, this study adds valuable information to the relatively small body of knowledge regarding real-life web-based health programs. However, there were also a number of limitations. Detailed data was only available for Facebook prior to and during the social media campaign, so it is not possible to assess the full impact of Instagram and display advertising on reach and raising awareness. It was also not possible to determine the exact numbers of new users who registered in response to the social media campaign since the usual opportunities to encounter and register with the program were available at the same time. At the time of the social media campaign, there was no “call to action” option for Instagram advertisements, which may have limited registrations numbers. It should be noted that the six weeks prior to the social media campaign included the Christmas period, which is known to be a time of lower engagement with the website.

## 7. Conclusions

The social media campaign resulted in a wide-reaching awareness of the 10,000 Steps program and increased numbers of new users. Notably, there were few differences in the way new users engaged with the program compared with other users, indicating that promoting the program this way is worthwhile. The SMC group succumbed to non-usage attrition earlier than the Year Prior group. As such, more research is needed to identify additional strategies, which can promote longer term engagement by users who register as a result of social media promotions. Facebook has been the main social media platform used for promotion of health programs in the past. However, given that Instagram achieved the greatest number of impressions and now has “click through” capacity, further research, examining the most effective combination of social media platforms for promotion and recruitment to health programs is warranted. This will assist in the designing of efficient, cost-effective, successful recruitment strategies for free web- and app-based health promotion programs.

## Figures and Tables

**Table 1:** Descriptive summary of demographic characteristics and engagement with the 10,000 Steps program, by social media campaign (SMC) and Year Prior groups and combined.

Characteristic/Engagement Parameter	Combined n = 1657	SMC Group ^a^ n = 1242	Same Period Year Prior to SMC Group ^b^ n = 415
	M (SD), n (%)	M (SD), n (%)	M (SD), n (%)
Age	44.1 (12.2)	44.6 (12.7)	42.6 (10.2)
Gender			
Female	1296 (78.2%)	996 (80.19%)	300 (72.3%)
Male	325 (21.61%)	245 (19.7%)	113 (27.23%)
Other	3 (0.18%)	1 (0.08%)	2 (0.48%)
Sessions	17.12 (30.86)	16.33 (32.06)	19.11 (27.56)
Pages viewed/session	9.10 (5.32)	9.03 (5.42)	9.30 (5.04)
Engaged with the website at least once	1398 (84.4%)	1000 (80.5%)	398 (95.9%)
Logged steps at least once	1104 (66.66%)	764 (61.5%)	340 (81.9%)
Average daily step count	9626 (4866)	9456 (4328)	10,006 (5885)
Average number of step entries	41.95 (43.92)	42.50 (48.66)	40.70 (30.79)
Challenges	62 (3.7%)	54 (4.3%)	8 (1.9%)
Tournaments	893 (53.9%)	573 (46.1%)	320 (77.1%)
Friend requests received and accepted	155 (9.4%)	112 (9.0%)	43 (10.4%)
Friend requests sent	107 (6.5%)	72 (5.8%)	35 (8.4%)

Notes: ^a^ SMC group = new Queensland 10,000 Steps users from social media campaign (21 January–3 March 2018); ^b^ Year Prior group = new Queensland users from the same time the previous year (21 January–3 March 2017).

**Table 2:** Total numbers of registrations and social media engagement and reach by time-periods relating to SMC ^a^.

SMC ^a^ Metric	Same Period Year Prior to SMC ^a^	6 Weeks Before SMC ^a^	During SMC ^a^
Overall registrations	1712	880	3141
QLD registrations	414	210	1242
All new FB page likes ^b^			
Total	48	42	461
Average daily	1.1	1.0	11.2
All FB reach ^c^			
Total	11,591	5266	391,131
Average daily	276.0	125.4	9312.6
All FB engagement ^d^			
Total	308	202	4952
Average daily	7.3	4.8	120.8
Instagram Impressions			819,192
Display Ad impressions			
Total			577,023
iOS			170,694
Android			95,434
Website Driver			310,885
Display Ad Clicks			
Total			1477
iOS			393
Android			329
Website Driver			755

Notes: ^a^ SMC = social media campaign (21 January–3 March 2018); ^b^ FB page likes = the number of new people who have liked 10,000 Steps Facebook Page; ^c^ FB reach = the number of people who had any content from, or about, the 10,000 Steps Facebook Page enter their screen. This includes posts, check-ins, ads, social information from people who interact with the 10,000 Steps Facebook Page and more; ^d^ FB engagement = the number of people who engaged with the 10,000 Steps Facebook Page, including any clicks or stories created.

**Table 3:** Costs, impressions, and reach associated with development and delivery of the 10,000 Steps program SMC ^a^.

Item	Total Cost	Cost per Click	Cost per Thousand Impressions ^b^	Clicks	Click through Rate ^c^
Total cost	$29,062.10				
Development costs	$4950.23				
Advertisement delivery	$24,111.87				
Facebook	$11,668.13 ^d^	$1.35	$37.16	8656	1.84%
Instagram ^e^	$5000.00	-	$6.11	-	-
Display ads	$7500.00	$5.11	$13.09	1477	0.26%

Note: ^a^ SMC = social media campaign; ^b^ Impression = a single view of an ad by one individual; ^c^ Click through rate = clicks per impression; ^d^ Facebook total costs include marketing promotion, adserving and optimization costs; ^e^ Instagram did not have a “call to action” option at the time of the social media campaign.

**Table 4:** Means and differences in daily registrations ^a^ to 10,000 Steps Program between time-periods relating to SMC ^b^.

Registration Period	Queensland Daily Signups		
	M (95% CI) ^e^	Between-Group Co-Efficient (95% CI)	*p* ^f^
Reference category: SMC ^b^ period	29.57 (23.15, 35.99)		
6 weeks prior to SMC ^c^	5.00 (3.03, 6.97)	−24.57 (−31.28, −17.86)	<0.001
Same period 1 year prior to SMC ^d^	9.86 (7.20, 12.51)	−19.71 (−26.66, −12.77)	<0.001

Notes: ^a^ Daily registrations in Queensland only; ^b^ SMC = social media campaign, n = 1242; ^c^ 6 weeks prior to SMC, n = 210, ^d^ Year prior to SMC, n = 415; ^e^ M (95% CI) = mean (95% confidence interval); ^f^ Alpha = 0.05; model based on generalized linear regression using negative binomial family and identity link (since data right skewed).

**Table 5:** Associations between groups ^a^ for participating in Challenges, Tournaments, and receiving and sending friend requests.

Reference Category: SMC ^b^ Group	SMC ^b^ Group vs. Year Prior Group	
	OR (95% CI)	*p*
Participating in individual Challenges ^c^ (Reference category: no)	0.43 (0.20, 0.91)	0.03
Participating in team Tournaments ^d^ (Reference category: no)	3.90 (3.03, 5.04)	<0.001
Receiving and accepting friend requests ^e^ (Reference category: no)	1.09 (0.75, 1.58)	0.67
Sending friend requests ^e^ (Reference category: no)	1.43 (0.93, 2.19)	0.10
Ever logging steps (Reference category: yes)	2.86 (2.17, 3.76)	<0.001

Notes: ^a^ Groups examined: SMC group and Year prior to SMC group; ^b^ SMC = social media campaign; ^c^ adjusted for Tournaments; ^d^ adjusted for Challenges; ^e^ adjusted for Tournaments and Challenges.

**Table 6:** Means and differences between groups ^a,b^ in website usage, step counts, and steps entries.

Engagement Metric	SMC ^c^ Group	Year Prior to SMC Group	Between-Group Co-Efficient	*p* ^e^
	M (95% CI) ^d^	M (95% CI) ^d^	M (95% CI) ^d^	
Sessions ^f^	16.27 (14.88, 17.67)	17.00 (14.88, 17.66)	0.73 (−1.11, 2.58)	0.44
Pages per session ^f^	9.02 (8.69, 9.35)	9.31 (8.69, 9.35)	0.29 (−0.28, 0.86)	0.31
Daily step count ^g^	9454.86 (9146.52, 9763.19)	10,008.37 (9384.92, 10,631.83)	553.52 (−143.58, 1250.62)	0.12
Total step entries	41.84 (38.87, 44.81)	40.07 (37.43, 42.71)	−1.77 (−5.61, 2.07)	0.37

Notes: ^a^ Groups examined: SMC group and Year prior to SMC group; ^b^ SMC group website usage, step counts and steps entries from 21 January to 31 December 2018, Year prior to SMC group website usage, step counts and steps entries from 21 January to 31 December 2017; ^c^ SMC = social media campaign; ^d^ M (95% CI) = mean (95% confidence interval); ^e^ Alpha 0.05; ^f^ model based on generalized linear regression using negative binomial family and identity link, adjusted for Challenges and Tournament counts; ^g^ model based on generalized linear regression using Poisson family and identity link, adjusted for Challenges and Tournament counts.

**Table 7:** Cox proportion hazard risks for non-usage-attrition, by group and by participation in Challenges and Tournaments.

Group/Challenge or Tournament Category	HR (95% CI)	*p*
Year Prior group ^a^	Reference	
SMC group ^b^ (unadjusted) ^c^	1.39 (1.24, 1.56)	<0.001
SMC group ^b^ (adjusted) ^d^	1.14 (1.01, 1.28)	0.03
Participated in Challenge	Reference	
Did not participate in Challenge	3.70 (2.82, 4.85)	<0.001
Participated in Tournament	Reference	
Did not participate in Tournament	3.02 (2.70, 3.37)	<0.001

Notes: ^a^ Year Prior group = new Queensland users from the same time as social media campaign period one year prior, n = 415; ^b^ SMC group = new Queensland users from social media campaign period, n = 1242; ^c^ unadjusted model; ^d^ model adjusted for participation in Challenges and Tournaments.

**Table 8:** Survival time by group of users of the 10,000 Steps Program (n = 1657).

User Group	Percentage of Group Still Using Platform		
	75%	50%	25%
SMC group ^a^	0 day	15 days	30 days
Year Prior group ^b^	7 days	29 days	43 days
Both groups combined	0 day	21 days	35 days

Notes: ^a^ SMC group n = 1242; ^b^ Year Prior group n = 415.

## Supplementary Materials

The following are available online at https://www.mdpi.com/1660-4601/16/24/5076/s1, Table S1: All daily data 2017–2019 incl app and website, Table S2: SMC and 2017 website usage, tournaments, etc., steps data, Table S3: Survival data.
